# Correlation analysis of sperm DNA fragmentation index with semen parameters and the effect of sperm DFI on outcomes of ART

**DOI:** 10.1038/s41598-023-28765-z

**Published:** 2023-02-15

**Authors:** KangSheng Liu, XiaoDong Mao, Feng Pan, YaJun Chen, Ruifang An

**Affiliations:** 1 Department of Obstetrics and Gynecology, The First Affiliated Hospital of Xi’an Jiaotong University, Xi’an, 710061 Shannxi China; 2 Department of Clinical Laboratory, Women’s Hospital of Nanjing Medical University, Nanjing Maternity and Child Health Care Hospital, Nanjing, 210029 Jiangsu China; 3 Department of Endocrinology, Affiliated Hospital of Integrated Traditional Chinese and Western Medicine, Nanjing University of Chinese Medicine, Nanjing, 210028 Jiangsu China; 4 Department of Andrology, State Key Laboratory of Reproductive Medicine, Women’s Hospital of Nanjing Medical University, Nanjing Maternity and Child Health Care Hospital, Nanjing, 210029 Jiangsu China

**Keywords:** Biochemistry, Biomarkers, Diseases, Urology

## Abstract

Routine semen analysis provides limited information about a man’s male reproductive potential and can not always fully explain male infertility. Many male infertilities are caused by sperm DNA defects that routine semen quality analyses fail to detect. In this study, we analyzed the association of sperm DNA fragmentation index (DFI) with the semen routine, sperm morphology, in vitro fertilization embryo transfer (IVF-ET)/intracytoplasmic sperm injection (ICSI). Further, we explored the predictive value of sperm DFI in evaluating male fertility and the outcome of IVF-ET/ICSI. Data on sperm DFI, sperm routine, and sperm morphology were collected from 1462 males with infertility. According to DFI levels, there were 468 cases in group I (DFI ≤ 15%), 518 cases in group II (15% < DFI < 30%), and 476 cases in group III (DFI ≥ 30%). The correlations of sperm DFI with semen routine and malformation rate were analyzed. Seminal plasma malondialdehyde (MDA), and total antioxidant capacity (TAC) were assessed. Sperm DFI, semen routine, and sperm morphology were detected in male patients of 101 pairs of IVF-ET/ICSI infertile couples and subdivided into IVF-I group (DFI ≤ 15%), IVF-II group (15% < DFI < 30%), IVF-III group (DFI ≥ 30%), ICSI-I group (DFI ≤ 15%), ICSI-II group (15% < DFI < 30%) and ICSI-III group (DFI ≥ 30%) according to DFI value. The effect of sperm DFI on the outcome of IVF-ET/ICSI was analyzed. There were significant differences in sperm survival rate, sperm concentration, and PR% between groupIII and group II (*P* < 0.01). There were significant differences in sperm survival rate, sperm concentration and PR% between group III and group I (*P* < 0.01). There was no significant difference in semen volume, age, abstinence days, or percentage of normal sperm between the three groups (*P* > 0.05). DFI was positively correlated with MDA content ( *P* < 0.01) and negatively correlated with TAC (*P* < 0.01). Sperm DFI was negatively correlated with sperm survival rate, sperm concentration, and PR% (*P* < 0.01). There was no correlation with age, abstinence days, semen volume, or percentage of normal-form sperm (*r* = 0.16, 0.05, 0.04, -0.18, *p* > 0.05). Compared with IVF-I group, the sperm concentration and PR were decreased in IVF-III group. The sperm malformation rate was higher in IVF-III group than that in IVF-II group. Comparatively, the PR was decreased in ICSI-III group. The sperm malformation rate was higher in ICSI-III group than that of the ICSI-I group (*P* < 0.05). There were no statistically significant differences in fertilization rate, cleavage rate, embryo rate, and clinical pregnancy rate between IVF group or ICSI group, and between all subgroups (*P* > 0.05). Sperm DFI is negatively associated with sperm survival rate, sperm concentration, and PR%. Antioxidants can decrease the rate of DNA fragmentation. Sperm DFI has proven to be very valuable in the male fertility evaluation, but its significance as a predictor of pregnancy outcomes following assisted reproductive technology. (ART) requires further investigation.

## Introduction

With the rapid development of the economy, the change in social structure, and the increasing life expectancy of human beings, the delay of childbearing age has become a universal phenomenon in the world^1^. Infertility is a complex reproductive disorder affecting nearly 15% couples, with 50% of cases being caused by male factor^2^. Routine semen analysis is the cornerstone of male laboratory work and the basic examination for male infertility diagnosis. Conventional semen analysis is mainly based on the microscopic cell characteristics of semen to assess its quality, including concentration, vitality, morphology, and other parameters. These conventional parameters provide basic information for the assessment of male fertility. However, routine semen analysis has its limitations in clinical application. On the one hand, routine semen parameters cannot accurately assess male fertility (about 15% of infertile men showed normal semen parameters)^3–5^. Moreover, the reference values of semen routine parameters are not entirely equivalent to the fertility assessment. Nor can it be defined as the minimum value for fertility**.** This suggests that semen analysis alone can only provide limited information for the assessment of male fertility, and it does not fully reflect the fertilization potential of the sperm.

With the development of reproductive medicine, it was found that Sperm DNA fragmentation (SDF) was produced in male germ cells during spermatogenesis and maturation periods, which can reflect the integrity of and the damage to the DNA. Damage to sperm chromatin can directly affect normal physiological functions and also leads to the transmission of incorrect genetic information to the offspring. Sperm DFI is a parameter that assesses male fertility^8,9^. Nowadays, sperm DFI has become an adjunct test for standard semen analysis. A lower conception rate may result from myriad factors, including increased SDF^6,7^. SDF impacts fertilization, embryonic development, etc. The damaged sperm DNA may lead to unsuccessful pregnancy outcomes, which could not be evaluated by the routine semen analysis. The simultaneous detection of the above-mentioned semen parameters can provide a comprehensive understanding of the patient’s semen status, sperm motility, sperm morphology, defect degree of genetic material in sperm head nucleus, and accessory gonadal secretion function, which has important guiding significance for the diagnosis and treatment of infertility. Although SDF has been increasingly evaluated in the clinical setting, it is not yet recommended as a routine test by the American Society for Reproductive Medicine^9^**. **In contrast, the American Urological Association (AUA) and the European Association of Urology (EWAU) have acknowledged the importance of DNA fragmentation in sperm as guidelines on male infertility^10^.

Sivanarayana et al. found that when the rate of sperm DNA fragmentation was higher than 30% in patients with ICSI cycle, the clinical pregnancy rate significantly decreased, and the abortion rate significantly increased^11^. The research results of Simon et al. in IVF cycles also showed that there was a significant negative correlation between the degree of sperm DNA damage and the live birth rate^12^. Conversely, a meta-analysis by Zini et al. suggested that sperm DNA damage has no effect on outcomes of ART pregnancies, though there was evidence that sperm DNA damage causes an increased risk of early abortion in IVF or ICSI pregnancies^13^. One reason for the inconsistency in published results is the variety of methods used to detect SDF in different studies. In this study, firstly, the relationship between sperm DFI and various sperm parameters was analyzed, and the diagnostic value of sperm DFI was evaluated by observing the semen parameters of infertile patients without gonadal accessory infection.. Subsequently, sperm DFI was detected by the SCD method after strictly controlling the influence of female factors and high sperm malformation rates in male patients. In addition, the value of sperm DFI in assessing male fertility and predicting the outcome of in vitro fertilization-embryo transfer (IVF-ET)/intracytoplasmic sperm injection (ICSI) were also investigated.

## Materials and methods

### Study design

There were 1462 infertile patients (one year of unprotected intercourses without pregnancy in a couple without detected female infertility factors); Ages ranged from 22 to 43 years, with a mean age of 31.6 ± 3.2 years who visited at the male department and reproductive Medicine Center at Women’s Hospital of Nanjing Medical University between August 2018 and September 2019 were included in the present study. The inclusion criteria are: (1) Sperm malformation rate ≤ 96%; (2) The female was ≤ 40 years old, body mass index (BMI) ≤ 30 kg/m^2^, and corresponding (blood routine, urine routine, reproductive hormone, serum anti-sperm antibody) tests were at normal levels. The semen test (mycoplasma, chlamydia, gonococcal) was negative. (3) FSH < 10.00 IU/L was detected in the outpatient department of our hospital for the first time. The cause of infertility was only the fallopian tube and the number of eggs obtained was ≥ 5^14^. (4) Both couples received IVF-ET or ICSI for the first time. Exclusion criteria : (1)The male partner had severe oligospermia (sperm concentration of the male partner < 5.00 × 10^6^ /mL). (2)Excluded were those with family diseases, genital trauma, and organic lesions, urological and reproductive diseases (e.g. varicocele, cryptorchidism, prostatitis, epididymitis, leukocytopenia, hematospermia, and azoospermia. (3)Excluded were those with diseases: endometriosis and Polycystic Ovary Syndrome (PCOS) (4) The male who has severe impairment**s** of heart, liver, and kidney function, smokers were excluded.. (5) Female genital malformation such as the double uterus, single horn uterus, mediastinal uterus, severe uterine cavity adhesion, endometrial thin and other conditions affecting embryo implantation. (6) The woman had thyroid disease, diabetes, and other endocrine diseases^15^. According to DFI levels, there were 468 cases in group I (DFI ≤ 15%), 518 cases in group II (15% < DFI < 30%), and 476 cases in group III (DFI ≥ 30%).

The IVF mainly consisted of couples with female factor infertility, and the criteria for ICSI was a total sperm count of < 800,000 after gradient centrifugation. A total of 101 pairs of IVF-ET/ICSI infertile couples were included in the study (including 56 cases of IVF and 45 cases of ICSI) and subdivided into IVF-I group (DFI ≤ 15%), IVF-II group (15% < DFI < 30%), IVF-III group (DFI ≥ 30%), ICSI-I group (DFI ≤ 15%), ICSI-II group (15% < DFI < 30%) and ICSI-III group (DFI ≥ 30%) according to DFI value**.**Among the 56 pairs in the IVF group, the male age was 22–43 years old (32.63 ± 5.21), and the female age was 25–40 years old (31.62 ± 4.05). In ICSI group of 45 pairs, the male age ranged from 26 to 51 years old (32.83 ± 5.62), and the female age ranged from 25 to 40 years old (31.63 ± 5.32). The present study was approved by the Ethics Committee of the Women’s Hospital of Nanjing Medical University and was conducted in accordance with the Declaration of Helsinki. An information sheet was provided to all participants. Written informed consent was obtained from all participants. The relevant guidelines and regulations of the local institute were strictly followed when conducting the study. Participants were informed that they could withdraw from the trial without giving a reason.

### Semen collection and routine semen analysis

According to the Laboratory Manual of the WHO for the Examination and Processing of Human Semen (5th edition) and WHO Manual for the Standardized Investigation, Diagnosis and Management of the Infertile Male^11^, semen samples were collected by masturbation after 2 to 5 days of ejaculatory abstinence^16^. The duration of abstinence was recorded. Each semen sample was directed into a sterile plastic cup and liquefied in an incubator at 37 °C. The semen liquefaction was complete, 10 μL of the sample was taken and counted, and sperm concentration and viability were recorded.

The routine semen analysis was performed with a semen quality detection system (CFT-920, Jiangsu Ruiqi Life Science & Tech Dev. Co.Ltd) with supporting reagents. The main parameters were as follows^17^. Image acquisition frame: low and middle sperm concentration collected at 20 Hz, and high sperm concentration at 7 Hz; acquisition interval: 3 ms; maximum sperm motile velocity: 200 μm s^−1^; area range of spermatozoa head detected at 7–60 μm^2^. Index of sperm motility: straight line velocity (VSL). Grayscale thresholds were set to collect spermatozoa and exclude nonsperm granules. According to the thresholds set for sperm analysis, sperm images were collected and analyzed. The sample was considered normal if semen volume had > 1.5 ml volume, ≥ 15 million/mL sperm concentration, ≥ 40% progressive motility and ≥ 4% normal morphology^18^^**.**^

### Sperm morphology assessment

For morphological evaluations, seminal smears were stained with Diff-Quik (MICROPTIC S.L. Co., Barcelona, Spain)^19^. Approximately 10 μl of sperm was smeared into a thin and homogeneous layer on a clean glass slide and was air-dried at room temperature for at least 10 min. The slides were stained and observed under a brightfield microscope (BH-2; Olympus, Tokyo, Japan) at 1000× magnification. According to WHO guidelines, a sperm with a deformed head, midpiece, or principal piece was counted as SDI (sperm deformity index), which is the number of deformed sperm/number of total sperm. For each semen sample, at least 200 sperms (or the whole sperm if the slide had less than 200 sperm) were counted via a double-blinded method. Then, the percentage of sperm with normal morphology was calculated^20^.

### Semen optimization

After the semen was completely liquefied, the sperm was selected by discontinuous density gradient centrifugation combined with the swimming-up method, as follows.

### (1) Discontinuous density gradient centrifugation

Two prepared gradient centrifugation media, one with 80% concentration and another with 40%, were preheated to 37 °C in in the incubator. In a sterile conical centrifuge tube, 1.5 ml of 80% gradient centrifuge medium was pipetted under 1.5 ml of 40% medium, and 2.0 ml semen was slowly added onto the top layer. After centrifugation at 300–400×*g* for 15 min, remove the supernatant, leaving only about 0.5 ml at the bottom. Then 2 ml Fertilization Medium was added to sperm deposition and thoroughly mixed. After one more centrifugation at 300–400×*g* for 5 min and removing of the supernatant, sperm deposition was transferred to a Falcon 1006 centrifuge tube containing 0.5 ml Fertilization Medium^18^.

### (2) Sperm swimming-up

The Falcon1006 centrifuge tube was tilted at 30—45 °C degree angle in a 37 °C incubator with 5% CO_2_ and saturated humidity. The process of sperm swimming-up lasted for half an hour, and then the upper sperm suspension was aspirated into another clean Falcon1006 centrifuge tube for later use^18^.

### DFI (SCD test)

To measure the DNA fragmentation in native and DGC-separated semen, the SCD test was performed using the SpermFunc™ DNAf kit (BRED Life Science, Shenzhen, China). Gelled aliquots of low-melting-point agarose in the kit were provided for semen sample processing in Eppendorf tubes. Eppendorf tubes were placed in a water bath at 80 °C for 20 min to melt the agarose and then transferred to a water bath at 37 °C for 5 min for temperature equilibration. A total of 60 μl of sampled semen was added to and mixed with the agarose in the Eppendorf tubes. Then, 30 μl of the semen-agarose mixture was pipetted onto precoated slides in the kit that were covered with a 22 × 22-mm coverslip. The slides were placed on a cold plate in the refrigerator (4 °C) for 5 min, allowing the agarose to produce microgel in which the sperm cells were embedded. The coverslips were gently removed, and the slides were immediately immersed horizontally in solution A and incubated for 7 min. Next, the slides were horizontally immersed in solution B for 25 min. After being washed for 5 min in a tray with abundant distilled water, the slides were dehydrated in gradient concentrations of ethanol (70%, 90%, 100%; respectively) for 2 min, air-dried, and stored at room temperature in opaque closed boxes^21^.

For bright-field microscopy, the slides were horizontally covered with a mixture of Wright’s staining solution (BRED Life Science, Shenzhen, China) and phosphate buffer solution (BRED Life Science, Shenzhen, China) (1:2) for 15 min with continuous airflow. Then, the slides were washed in running water for 10 s and allowed to dry. Intense staining was recommended to allow the periphery of the dispersed DNA loop halos to be more visible. A minimum of 500 sperm were counted on each sample under the 100× magnification^22^**.**

**Figure 1:**
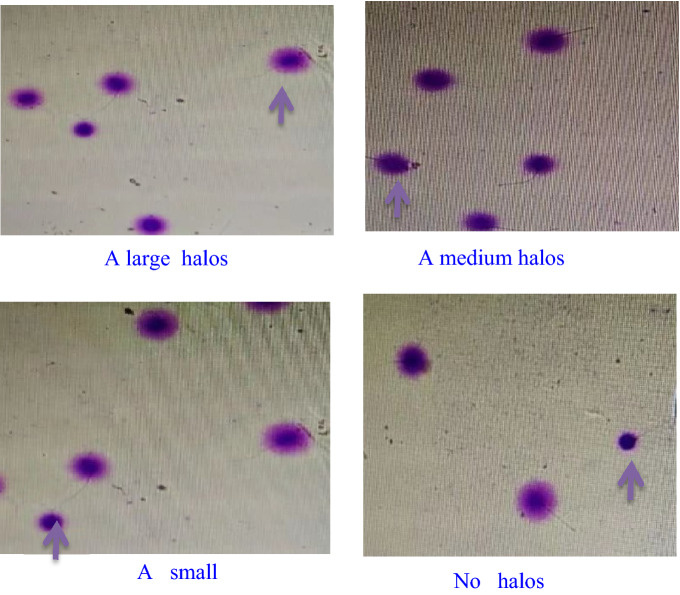
SCD test of human sperm.

Normal spermatic DNA presented radiate halos, and damaged spermatic DNA presented no or small halos. Fragmented sperm refers to those having a small or no halo (Fig. 1). The thickness of the halo on one side was less than the 1/3 diameter of the head’s thinnest part^23^. The rate of SDF (%) = the number of sperm with fragmented DNA/the totalnumber of sperm × 100%, and < 25% was considered normal.

### Quantitative detection of seminal plasma biochemical indexes

Spectrophotometry was used to test TAC (U/L) and MDA (nmol/mL) levels. MDA levels were determined using the thiobarbituric acid (TBA) method. Semen samples were centrifuged at 4 °C for 15 min with a speed of 2000 r/min. The supernatant was mixed with the reagents supplied in an MDA Assay Kit (Nanjing Jiancheng Bioengineering Corporation, China, A003-2) and incubated at 95 °C for 40 min. Having been cooled at room temperature, the mixture was centrifuged at 4000*g* for 10 min. The absorbance of the supernatant was measured at 530 nm. All operations were performed according to the manufacturer’s instructions. The MDA concentrations were expressed as nmol/mL^24^.

### Treatment of IVF-ET and ICSI

The woman followed a regular long regimen of gonadotropin-releasing hormone agonist (GnRH-α). The follicle development was monitored by vaginal B-ultrasound and serum estradiol (E2) level. After follicle maturation, human chorionic gonadotropin (HCG) was injected intravascularly. Egg extraction was performed 36 h later under the guidance of B-ultrasound. Fertilization was observed 16–18 h after insemination. Blastocysts were rated by the Gardner method, and D5 score ≥ 3BB or D6 score ≥ 4BB were defined as high-quality embryos. One to two high-quality embryos were frozen and thawed for transplantation, and luteal support was performed after transplantation. 35 days after transplantation, b-mode ultrasonography showed that the primary heart tube beating of the gestational sac was clinical pregnancy^25^.

### Oocyte fertilization and embryo culture and transfer

For IVF fertilization, fresh semen was subjected to DCG in sperm-grade 40% and 80% solutions for pretreatment, followed by the sperm swim-up technique to adjust to a final sperm density of 1 × 10^6^/ml. The solution was then cultured with oocytes for fertilization. In ICSI fertilization, after DCG of the sperm from fresh semen, viable sperm with good morphology were collected under the microscope and directly injected into the egg cytoplasm for fertilization^26^. The pronuclei of the oocytes were examined 16–18 h after injection to assess the success of fertilization^27^.

### Calculation of IVF/ICSI clinical outcomes

$$\begin{gathered} {\text{IVF\, fertilization\, rate }} = {\text{ the \,number\, of \,fertilized \,eggs\,}}/{\text{\,total \,number\, of\, obtained \,eggs}} \times {1}00\% . \hfill \\ {\text{Fertilization\, rate\, of \,ICSI }} = {\text{number\, of \,fertilized\, eggs\,}}/{\text{number\, of\, M\,}} {\text{II eggs}} \times {1}00\% . \hfill \\ {\text{Cleavage \,rate }} = {\text{the \,number\, of \,fertilized\, cleavage\, embryos}}/{\text{number \,of \,fertilized\, eggs}} \times {1}00\% . \hfill \\ {\text{High-quality\, embryos\, rate }} = {\text{the \,number \,of\, high-quality\, embryos\,}}/{\text{\,number\, of\, normally\, fertilized \,embryos }} \,\times \,{1}00\% . \hfill \\ {\text{Clinical\, pregnancy\, rate }} = {\text{ the\, number\, of \,clinical\, pregnancy\, cycles }}/{\text{\,the \,number \,of\, all\, transplant\, cycles}} \times {1}00\% . \hfill \\ \end{gathered}$$

### Statistical analysis

Statistical analyses were performed by with SPSS software Version 22.0 (SPSS Inc., Chicago, IL, USA) for Windows. The measurement data were tested for normality and homogeneity of variance. Data that do not conform to normal distribution and homogeneity of variance will be represented by median (P25, P75). Kruskal–wallis H test was used for comparison between groups, the Nemenyi test was used for pial comparison, and Spearman’s test was used for correlation analysis. Data consistent with normal distribution and homogeneity of variance were expressed by mean ± standard deviation. Comparison between groups was performed by one-way ANOVA, SNK-q test was used for pairwise comparison. Counting data were presented as cases or cases (%), and comparison between groups was performed by χ^2^ test, *P* < 0.05 was considered statistically significant.

## Results

### Comparison of semen parameters between different sperm DFI levels

When compared with group II (between group III and group II), there were significant differences in sperm survival rate, sperm concentration, and PR% (*P* < 0. 01). There were no significant differences in age, abstinence days, semen volume, percentage of normal sperm (*P* > 0. 05). There were significant differences in sperm survival rate, sperm concentration, and PR% between group III and group I (*P* < 0. 01).There was no significant difference in semen volume, in age, abstinence days or percentage of normal sperm (*P* > 0. 05). DFI was positively correlated with MDA content (*P* < 0.01) and negatively correlated with TAC (*P* < 0.01), see Table 1.

**Table 1:** Comparison of semen parameters between different sperm DFI levels.

	I	II	III
Parameter	M (*P*25,*P*75)	M (*P*25,*P*75)	M (*P*25,*P*75)
Abstinence days (days)	4.1 (3.1, 5.8)	3.9 (3.1, 7.3)	4.8 (3.6, 8.1)
Age (years)	28.9 (23.0, 33.0)	36.0 (23.0, 39.3)	36.1 (26.1, 39.6)
Sperm survival rate (%)	41.3 (34.2, 50.1)	35.6 (31.5, 43.7)*	27.7 (18.6, 38.5) *#
Semen volume (ml)	3.0 (2.2, 4.3)	3.05 (2.3, 4.2)	3.5 (3.5, 4.7)
Sperm concentration (× 10^6^/ml)	85.5 (60.3, 150.3)	66.2 (56.3, 89.6)*	45.5 (21.6, 61.5)*#
Normal sperm (%)	2.1 (1.0, 3.2)	2.0 (1.1, 4.6)	1.5 (0.6, 1.7)
PR%	36.1 (30.3, 42.5)	28.8 (23.5, 37.3)*	21.3 (10.6, 32.3)*#
Sperm deformity rate (%)	97.00 (95.10, 99.10)	98.00 (96.00, 100)	99.31 (96.10, 100)
MDA (nmol/ml)	5.13 (3.21, 6.30)	7.11 (5.82, 9.15)	9.65 (6.92, 11.63)
TAC (U/L)	20.52 (16.60, 22.16)	15.50 (13.21, 18.30)	11.35 (9.51, 15.15)

All data in the table are represented by M (P25, P75). * Compared with group I, # compared with group II, *P* < 0.01.

According to DFI levels, there were 468 cases in group I (DFI ≤ 15%), 518 cases in group II (15% < DFI < 30%), and 476 cases in group III (DFI ≥ 30%).

### Correlation analysis between sperm DFI and semen parameters

Sperm DFI was negatively correlated with sperm survival rate, Sperm concentration, and PR%. There was no correlation with age, abstinence days, semen volume, or percentage of normal sperm.

### The basic characteristics, semen parameters, and malformation rates of the three subgroups were compared

The comparison of clinical data among subgroups is shown in Table 3. Compared with the IVF-I group, the IVF-III group had lower sperm concentration and PR. The rate of sperm malformation in the IVF-III group was higher than that in the IVF-II group. Compared with the ICSI-I group and ICSI-II group, ICSI-III PR was decreased. The sperm malformation rate of the ICSI-III group was higher than that of the ICSI-I group. There were no significant differences in female age, BMI, FSH, number of eggs obtained, and male age in each subgroup of IVF and ICSI groups.

### Clinical outcomes of different subgroups

Comparison of clinical outcomes among subgroups, there were no significant differences in fertilization rate, favorable embryo rate, clinical pregnancy rate, and cleavage rate between the IVF group and ICSI group, as shown in Table 4.

## Discussion

Infertility is one of the world’s most important public health and clinical problems. Due to the social environment, psychological pressure, the delay of childbearing age, and other factors, the proportion of infertile couples has increased accordingly, and assisted reproductive technology has also developed rapidly. However, the diagnostic criteria for male infertility are mainly the traditional semen parameters recommended by the World Health Organization (WHO). An increasing number of studies explored new biological indicators with crucial clinical significance. Spermatozoa DFI is proven as an important supplement to semen routine assessment, which elevates semen routine assessment from concentration, quantity, morphology, and other levels to the molecular level. It has been reported that high DFI can have adverse effects on semen quality and ART outcome^28^, but the correlation between sperm DFI and age, semen routine parameters, abnormality rate, and its effect on ART outcome has been debated. In this study, sperm DFI was detected by the SCD method after strictly controlling the influence of female factors and high sperm malformation rate in male patients, followed by exploring the importance of sperm DFI in evaluating male fertility and predicting IVF-ET/ICSI outcome.

### Correlation between sperm DFI and age, semen routine parameters, MDA content, and TAC levels

This study showed that sperm DFI level was negatively correlated with sperm survival rate and PR% of statistical significance, which is consistent with relevant research reports^29–31^, and there were statistical differences among groups I, II, and III. The sperm survival rate and PR% decreased significantly with the increase of DFI level does not mean the relatively low percentage of the former indicated the damage of sperm DNA. However, abnormal sperm DNA affects not only sperm fertilization ability but also inhibits embryo development. However, most PR% values of these two groups were below the lower limit of WHO reference value^32^, cases of which were classified as asthenospermia^33^. In this study, inflammation and infection have been excluded, which may be related to the endocrine function of the seminal vesicle and other factors. In summary, the results showed that the percentage of normal sperm in group III was statistically different from that in group II, which was consistent with some reports^16^, and the former was significantly lower than the latter. Although there was no statistical difference between group I and the other two groups, the percentage of normal sperm was higher than that of group III and lower than that of group II. In this study, no correlation was found between patient age, abstinence days, semen volume, sperm concentration, and sperm DFI (Tables 1 and 2). However, it can be seen from the Table 1 that DFI increases with age, and it has been reported that age is positively correlated with DFI (With the aging of male organs such as the testis, prostate, and epididymis, it leads to the increase in the reactive oxygen species (ROS) and the decline of antioxidant capacity^34,35^. Too much ROS produces many lipid peroxides, which attack the membrane of sperm cells, causing the sperm DNA strand to break and destroy its integrity)^36^_._ About 60% of the seminal plasma comes from the seminal vesicle, 30% from the prostate, and the rest from the epididymis, paraurethral gland, paraurethral gland, testis, etc.^37–40^.

**Table 2:** Correlation analysis between sperm DFI and semen parameters.

Parameter	*r*	*P*	Parameter	*r*	*P*
Abstinence days	0.05	0.43	PR%	−0.43	0.002
Age	0.16	0.07	Sperm survival rate	−0.55	0.003
Sperm concentration	−0.15	0.03			
Semen volume	0.04	0.46			
Percentage of normal sperm	−0.18	0.08			

Many studies have proposed hypotheses on the mechanism of sperm DNA damage. The main possibilities are as follows: (1) ROS may cause the rupture of sperm DNA chain through direct oxidation of sperm DNA bases or covalent binding of lipid peroxidation products and DNA, leading to damage to the biological structure of sperms or DNA damage, (2) abnormal sperm chromatin assembly can lead to sperm DNA double-strand break. Three factors are currently considered to be the main cause of sperm damage, including abnormal sperm chromatin assembly, aberrant sperm cell apoptosis, and excessive oxidative stress^41^. During sperm maturation, histones are gradually replaced by the arginine-rich, cysteine-rich, and smaller protamine (HP), which causes the reduction of sperm DNA self-repair ability in response to internal changes and the external environment. Furthermore, under the effect of torsion-tension generated by the double-stranded DNA helixes, the misfolding of DNA supercoiling structures in chromosomes can also lead to aberrant DNA repair, which results in abnormalities of the chromatin structures and an increase in SDF^42–46^^.^ Therefore, sperm DFI can be detected to reflect the defection degree of genetic material in the sperm nucleus and sperm DNA maturity status. In this study, the low DFI group had an obviously lower seminal MDA level and an obviously higher seminal TAC level than high DFI groups, indicating that too much MDA was produced during seminal lipid peroxidation and that the drop of TAC level triggered oxidative stress reaction and destroyed the spermatic membranes^47^. According to Ni et al.^48^ and Fu et al.^49^, ROS could cause sperm DNA damage in patients with varicocele. Shang et al^50^ and Greco et al^32^ have reported that antioxidants can decrease the rate of DNA fragmentation, suggesting that the seminal ROS participates in the process of sperm DNA damage.

### Relationship between sperm DFI and IVF/ICSI clinical outcome

This study showed no statistically significant differences in fertilization rate, cleavage rate, favorable embryo rate, and clinical pregnancy rate between the IVF group and ICSI group, which was consistent with the results of previous studies^51,52^. However, Simon et al.^53^ showed that higher sperm DFI would reduce IVF/ICSI clinical pregnancy rate via meta-analysis. Possible reasons for the results of this study are as follows:

1. In embryo transfer, high-quality embryos are preferentially selected, which may also be why sperm DFI is not related to clinical pregnancy outcomes.
2. Sperm DFI and abnormality rate reduced after sperm was optimized by upstream method and density gradient centrifugation so that the influence of high sperm DFI on clinical pregnancy outcome was weakened.
3. Early embryo development is controlled by maternal genes, sperm DNA damage is partially repaired after a successful pregnancy, late paternal gene activation plays a role, and patients with high sperm DFI embryos may be ultimately unable to continue the pregnancy to 3 months or final production. Cissen et al.^54^ also believe that there is insufficient evidence to recommend the routine use of SDF testing to predict pregnancy and select treatment in couples undergoing ART, and further studies are needed on the predictive value of sperm DFI for pregnancy rate after 3 months. The results were compared with other predictors of pregnancy after 3 months, such as female age, male age, semen parameters, and oocyte number. Esteves et al.^55^ also proposed the endpoint of the birth fragmentation test and suggested that high sperm DFI led to lower birth rates in IVF and ICSI groups. Sperm DFI is correlated with age, sperm concentration PR, sperm motility rate, and deformity rate. It is suggested that sperm DFI be used as a standard item in semen analysis to improve the evaluation of male fertility and guide the treatment. Sperm DFI has no predictive value for IVF/ICSI clinical outcome, and it is not recommended to use sperm DFI as an examination item in the selection of ART regimen (Tables 3 and 4). Bronet et al.^56^ performed chromosome aneuploidy analysis on 154 embryos from 38 patients treated with PGD due to repeated abortions or repeated implant failures. By analyzing the relationship between sperm DFI and embryo chromosome status, no evidence had been proven that SDF was a risk factor for aneuploidy. The prospective study results of Esbert et al.^57^ showed that SDF did not affect clinical pregnancy outcomes of patients undergoing IVF or ICSI treatment using autologous or donor eggs. A growing number of studies have also shown that the sperm of infertile men with a high degree of DNA damage can still reproduce successfully with assisted reproduction^58^.

**Table 3:** Comparison of basic characteristics, semen parameters and malformation rate between three subgroups.

Parameter	IVF-I (*n* = 23)	IVF-II (*n* = 21)	IVF-III (*n* = 12)	*χ*^2^ or *F*	ICSI-I (*n* = 12)	ICSI-II (*n* = 15)	ICSI-III (*n* = 18)	*χ*^2^ or *F*
Female age (year)	32.00 ± 4.69	31.61 ± 4.72	32.85 ± 5.66	0.285	32.16 ± 4.97	31.75 ± 4.80	32.66 ± 4.02	0.503
BMI (kg/m^2^)	23.52 ± 3.01	22.06 ± 6.21	23.50 ± 3.36	0.551	23.01 ± 3.41	22.58 ± 3.52	24.02 ± 4.61	1.515
FSH (U/L)	5.89 (5.51,9.26)	6.83 (6.26,9.15)	5.90 (5.02,8.63)	2.205	7.82 (5.95,9.01)	8.3 (6.86,10.22)	6.86 (5.60,7.90)	4.052
Number of retrieved oocytes (piece)	12.00 (7.61,18.08)	12.00 (9.00,15.00)	12.00 (9.80,15.00)	0.519	11.0 (7.00,18.00)	11.0 (4.80,18.90)	12.00 (9.00,17.00)	0.819
Male age (year)	32.30 (29.00,39.00)	33.00 (30,38.00)	38.00 (31,41.50)	2.133	34.0 (29.60,39.51)	32.0 (30.20,35.0)	31.80 (30.20,36.00)	0.624
Sperm deformity rate (%)	94.95 (94.00,96.00)	94.94 (93.00,96.00)	96.00 (96.00,96.00)#b	5.321*	95.1 (95.00,96.00)	95.92 (94.8,96.0)	95.97 (96.00,96.00)#c	8.263*
PR (%)	37 (26.21,41.22)	28 (15.50,32.80)	20 (19.65,35.05)#a	11.826*	38.20 (26.88,51.20)	26.11 (16.58,33.81)	19.52 (12.62,28.71)#cd	10.286*
Sperm concentration (× 10^6^ /mL)	89.10 (63.33,153.80)	59.20 (31.78,85.60)	45.30 (30.23,61.85)#a	5.162*	67.0 (35.8,105.29)	44.5 (16.80,67.55)	41.2 (20.60,99.17)	4.132
DFI (%)	11.90 (9.20,13.21)	18.63 (16.91,23.55)#a	38.35 (35.00,61.10)#a#b	55.208**	12.34 (8.01,14.59)	22.6 (17.31,25.0)#c	40.6 (35.21,52.87)#c#d	38.26**

**P < 0.01, *P < 0.05; Data in the table are expressed as ${\overline{{x}}}$  ± S or M (P_25_, P_75_).

#a: compared with IVF-I group, #b: compared with IVF-II group, #c: compared with ICSI-I group, #d: compared with ICSI-II group, *P* < 0.05.

According to DFI levels, IVF group was subdivided into IVF-I group (DFI ≤ 15%), IVF-II group (15% < DFI < 30%), IVF-III group (DFI ≥ 30%); ICSI group was subdivided into ICSI-I group (DFI ≤ 15%), ICSI-II group (15% < DFI < 30%) and ICSI-III group (DFI ≥ 30%).

**Table 4:** Comparison of clinical outcomes between different subgroups.

Parameter	IVF-I	IVF-II	IVF-III	*χ*^2^	ICSI-I	ICSI-II	ICSI-III	*χ*^2^
Number of transplant cycles	23	21	12	–	12	15	18	–
Number of retrieved oocytes	381	402	106	–	160	161	246	–
Number of MLL oocytes	263	281	88	–	130	125	212	–
Normal number of fertilized oocytes	241	238	71	–	101	102	147	–
Fertilized oocytes	319	326	85	–	112	115	179	–
Fertilization cleavage, embryo number	305	320	81	–	109	112	176	–
Fertilization rate (%)	319/381 (83.72)	326/402 (81.09)	85/106 (80.20)	3.256	112/160 (70.00)	115/161 (71.42)	179/246 (72.76)	5.622
Optimal rate of embryo (%)	120/241 (49.79)	112/238 (47.06)	31/71 (43.66)	0.261	33/101 (32.67)	41/102 (40.19)	62/147 (42.18)	5.636
The cleavage rate (%)	305/319 (95.61)	320/326 (98.16)	81/85 (95.29)	5.218	109/112 (97.32)	112/115 (97.39)	176/179 (98.32)	5.012
Clinical pregnancy rate (%)	12 (60.00)	9 (50.00)	4 (50.00)	0.856	6 (66.6)	5 (41.67)	7 (46.67)	1.803

The data in the table is represented by example or example (%). According to DFI levels, IVF group was subdivided into IVF-I group (DFI ≤ 15%), IVF-II group (15% < DFI < 30%), IVF-III group (DFI ≥ 30%); ICSI group was subdivided into ICSI-I group (DFI ≤ 15%), ICSI-II group (15% < DFI < 30%) and ICSI-III group (DFI ≥ 30%).

## In conclusion

In summary, our present study showed that Sperm DFI is correlated with age, sperm concentration, PR, sperm motility rate, and deformity rate. Sperm DFI can be used to evaluate male fertility effectively. However, Sperm DFI has proven to be very valuable in the male fertility evaluation, but its significance as a predictor of pregnancy outcomes following ART requires further investigation.

## Data Availability

The datasets used are analyzed during the current study and are available from the corresponding author on reasonable request.

## Abbreviations

ART: Assisted reproductive technology
IVF: In vitro fertilization
MDA: Seminal plasma malondialdehyde
TAC: Total antioxidant capacity
VSL: Straight line velocity
SH: Sulfhydryl
IVF-ET: In vitro fertilization-embryo transfer
ICSI: Intracytoplasmic sperm injection
GnRH-α: Onadotropin-releasing hormone agonist
TAC: Total antioxidant capacity
AUA: American Urological Association
EWAU: European Association of Urology
SDF: Sperm DNA fragmentation
BMI: Body mass index
ROS: Reactive oxygen species
